# Nurse-Training in Small Hospitals

**Published:** 1918-12-21

**Authors:** 


### THE MATRONS' AND SISTERS' DEPARTMENT.

### NURSE-TRAINING IN SMALL HOSPITALS.

### Its Troubles and their Solution.

This subject is felt by matrons and nurses to be one of pressing importance just now. It was mentioned at the Conference of Matrons on the occasion of the annual meeting of the College of Nursing, and a meeting on the subject was held at Brompton Hospital on December 12. On the latter occasion Miss Gibson suggested that small conferences should be held on this question. This should be very helpful. Many matrons who hesitate about addressing large numbers of strangers would be well able to express their ideas, if they were sitting round a table, with not more than six people in the room. The result could be given by one good speaker to larger meetings.

The number of suggestions at Conferences rarely equals that of complaints of the present system. On this particular question, one hears first the oft-repeated lament that matrons of large training schools object to taking probationers with previous experience in small hospitals. The matrons never really explain what they feel about it. The trouble is the varying standard of training in small hospitals. Whereas in some of these the nurses are excellently taught, both in theory and practice, in others they learn no theory at all, and in practice have a great deal to unlearn. Until this state of things is altered, it would be impossible to take these probationers as second-year nurses, as is often suggested. The scheme of a regular affiliation would cure this trouble as well as others. If the small hospital regularly passed its nurses on to the large one, or received its nurses from thence, for a year's training, its standard would necessarily rise: it must come into line. The nurses would be expected to have learned a definite amount, both of theoretical and practical nursing, during their time in the small hospital, and regular arrangements would be made for teaching them. At present, too much depends on the individual matron and doctor. If they are keen and conscientious, the nurses learn a great deal; but if not, there is no regular teaching, and probationers only learn what they pick up for themselves.

If affiliation is to be done at all, it ought to be done boldly, and on a large scale. All small and special hospitals, without exception, should be affiliated to the large training schools, on a business basis. With regard to nursing homes, they need not be considered, as probationers should never be taken in such establishments at all. For the heavy fees that are charged in them, the patients should be able to command skilled nursing.

The selection of probationers under an affiliation scheme is a difficulty. It would be best for the large school to supply the small one, because its choice of candidates is so much better both in quantity and quality. All matrons of small hospitals complain of the great difficulty which they experience in filling vacancies. They admit that the class of probationers which they train is not satisfactory, but say, quite truly, that they must use the material at hand, or in plain terms, take what they can get. Even in normal times the best candidates will naturally apply to the large training schools.

Many of the best matrons are strongly of opinion that the first year should be spent in the large school. It is very difficult, in small hospitals, to teach hospital etiquette, to keep up a good standard of discipline, and to instil the spirit of loyalty to the school. All these things should be learned in the first year. It is also hard on the small hospital to have a succession of first-year probationers who are sent off to the large hospital directly they begin to be of any use.

The objection usually put forward is that second-year nurses object to doing junior probationers' work. If this means that they do not wish to waste their time in scrubbing and cleaning brass, their point of view is extremely reasonable. It is very desirable that nurses should understand all kinds of domestic work; but they should study it before they come to hospital at all, either at home or at school. If, on the other hand, junior nurses' work means any kind of actual nursing, the second-year nurse should have no objection to doing it for the first part of her year in the small hospital. Cleaning babies' bottles in one hospital, or washing and attending to helpless patients in another, is real nursing, though of a simple kind, and no sensible probationer should make any difficulty about doing either. She would do more advanced work in the latter part of her term of training. The probationers in the small hospital would of course not all be moved at once. The change would take place say every six months. This makes the theoretical teaching rather difficult, but with small numbers the classes will be more like private coaching than lectures, and will gain very much in thoroughness. It is much easier to be sure that six nurses are attending to the subject in hand, and understanding what is told them, than to hold the attention of sixty, some of whom will perhaps wish to go to sleep or to draw caricatures of the lecturer, instead of taking notes.

Any scheme of affiliation must have its own difficulties and drawbacks. But if the goodwill of the matrons is gained, their practical wisdom will conquer all difficulties, for then the training of nurses in small hospitals can be placed on a permanently satisfactory footing.

